# Exploring the relationship between social media dependence and internet addiction among college students from a bibliometric perspective

**DOI:** 10.3389/fpsyg.2025.1463671

**Published:** 2025-03-12

**Authors:** Ruotong Dong, Dongfeng Yuan, Xue Wei, Jingyi Cai, Zhongzhu Ai, Shiquan Zhou

**Affiliations:** Faculty of Pharmacy, Hubei University of Chinese Medicine, Wuhan, China

**Keywords:** social media, college students, internet addiction, bibliometric, VOSviewer, systematic review

## Abstract

**Background:**

Social media use among college students often leads to psychological dependence, resulting in a rising number of internet addictions. The link between social media dependence and addiction is garnering increasing attention.

**Objective:**

The aim of this study was to analyze and discuss the research trends and hotspots on social media dependence and internet disorder among college students by bibliometric methods.

**Methods:**

Relevant studies on social media dependence and online addiction among college students were retrieved from the Web of Science (WoS) database spanning the years 2013 to 2024. We delineated the distribution of publications to identify the core productivity within the field. VOSviewer software was employed to conduct network visualization analyses of countries, authors, journals, and keywords, aiding in a comprehensive understanding of the research trends and hotspots in this domain.

**Results:**

From the WoS database, we retrieved 302 publications, and 167 publications were included after screening. The findings revealed: (1) a steady increase in publications and citations, particularly accelerating after 2019. (2) The most productive journal is *Computers in human behavior*, the most productive research area is Psychology, and the most productive author, institution, and country are Professor Griffiths MD, Nottingham Trent University and China, respectively. (3) Collaborative network analysis indicated that there were multiple research groups in this field, yet the connections among countries and authors remain relatively limited. (4) Co-citation analysis of journals revealed that this field was interdisciplinary, primarily integrating psychology, psychiatry, and behavioral science. (5) Keyword analysis identified two major research hotspots: the relationship between college students’ social media dependence and internet addiction, and the mediating factors influencing college students’ social media dependence and internet addiction. The mechanism of internet addiction is an emerging research frontier.

**Conclusion:**

This analysis outlines the progress and directions of research on college students’ social media dependence and internet addiction. It offers a comprehensive examination of the latest frontiers and trends, providing theoretical support for regulating the use of electronic products and implementing mental health interventions in college students.

## 1 Introduction

Recently, the proportion of global internet users has experienced exponential growth (Shakeri Hossein Abad et al., 2021). According to relevant survey reports, as of January 2024, the number of global internet users reached 5.35 billion, accounting for 66.2% of the world’s population, with 5.04 billion active social media users, constituting 62.3% of the global population (Petrosyan, 2024). The expansion of internet access has transformed the way people live, work, communicate, and learn, and has become an important medium for the rapid development of various industries (Pinho et al., 2021). Social media refers to content production and exchange platforms on the internet based on users’ social relationships. With the popularization of the internet, social media has also been widely applied in our daily lives. The extensive use of social media enables individuals to communicate opportunistically with both broad and narrow audiences and to consciously self-present, deriving value from user-generated content and the sense of connection with others (Carr and Hayes, 2015). Social media includes many applications and online channels, such as Facebook, YouTube, WeChat, QQ, and others. These programs and websites provide us with more opportunities to interact and collaborate with others, share useful and interesting content, and find entertainment to alleviate psychological distress (Elphinston et al., 2022). The convenience of mobile electronic devices allows people to access social media almost anytime and anywhere (Yılbaş and Günel, 2022), and most people tend to spend more time and effort on social media. Undoubtedly, social media plays an increasingly important role in today’s society and has become an indispensable part of people’s daily lives.

College students constitute a significant portion of social media users, and their reliance on social media has a profound impact on their susceptibility to internet addiction. Students aged 18–29 years exhibit a social media usage rate of 90%, which is substantially higher than the average for other age groups (Lau, 2017). Moreover, university represents a critical transitional period for students as they move from the family environment to broader society. During this phase, students need to establish extensive social networks to expand their interpersonal relationships, access information, and integrate into society. Social media provides them with convenient tools for communication and interaction (Ellison et al., 2007). College students often face multifaceted pressures related to academics, employment prospects, and self-identity. As a result, social media has become a key avenue for alleviating stress and seeking emotional support. However, due to their relatively immature psychological development, students are vulnerable to excessive usage when confronted with the vast array of information and features offered by social media (Kuss and Griffiths, 2011). Notably, during the COVID-19 pandemic, students’ lifestyles underwent significant changes, profoundly affecting their online behaviors and potential for addiction. For example, Li et al. (2021) found that, compared to the pre-pandemic period, students’ daily screen time increased by approximately 3–4 h. Ma and Sheng (2023) observed that students are more likely to visit entertainment websites or engage in online chats during class, which not only impairs their academic performance but also increases their overall screen time, potentially leading to a shift from habitual use to addiction. An increase in the time spent on social media and the diversification of its usage purposes heighten the risk of internet addiction (Balcı and Baloğlu, 2018; Mansi and Levy, 2013). Traditionally, addiction is defined as the repeated use of a substance despite experiencing negative consequences (Alavi et al., 2012). Similar to substance addiction, behavioral addiction is considered a habitual impulse or compulsion (Avena et al., 2008; Roberts et al., 2014). Some scholars have noted that addictive behaviors can reduce students’ sleep efficiency and prosocial behaviors, foster feelings of inferiority, lower life satisfaction, and contribute to poor academic performance (Sümen and Evgin, 2021; Hawi and Samaha, 2016; Azizi et al., 2019). Furthermore, such behaviors pose risks to mental health, including anxiety, burnout, and other psychological issues (Boer et al., 2021; Vannucci et al., 2017; Liu and Ma, 2020). Therefore, it is essential to gain a more comprehensive understanding of the relationship between social media dependence and internet addiction among college students.

Bibliometric analysis provides a quantitative approach to identifying research trends, key topics, and core issues within a specific field. In contrast to traditional qualitative reviews, bibliometric methods enable data-driven analysis that uncovers research patterns, knowledge structures, collaboration networks, and emerging topics, offering theoretical insights that can guide the future development of the field (Chen et al., 2024). In the present study we integrates bibliometric analysis with thematic summaries to address both quantitative trends and qualitative insights within the field. While bibliometric methods systematically map publication patterns, authorship networks, and citation dynamics, thematic interpretation delves into the conceptual underpinnings of research hotspots, offering a nuanced understanding of theoretical and practical implications. This dual approach ensures a comprehensive exploration of the research landscape, balancing macro-level trends with micro-level content analysis to enhance the depth and applicability of findings. This study aims to more objectively and comprehensively reveal the relationship between social media dependence and internet addiction among college students by bibliometric analysis and thematic summaries. These findings will offer valuable theoretical support for scholars in this field and other related fields, and help to carry out subsequent research.

## 2 Methods

### 2.1 Data collection and search strategy

In this study, four main databases were considered: WoS, PubMed, Scopus, and Google Scholar. Upon searching, it was observed that the WoS database contained the largest volume of literature. Following screening, removal of duplicates, and elimination of low-quality literature, the primary source of literature included in the analysis remained the WoS database. Given the higher quality and accuracy of citations within the WoS database compared to others, along with its status as one of the most standard and widely utilized databases in bibliometric analysis (Li et al., 2022), this study selected the WOS database as the primary source for literature collection.

The literature search strategy was as follows: firstly, to consider the impact of social media, the search terms were limited to TS = (“social media” OR “social network” OR “SNS” OR “online community” OR “forum” OR “Reddit” OR “Twitter” OR “Tweet” OR “Facebook” OR “Instagram” OR “Weibo” OR “Tumblr”). Secondly, the research subjects were limited to college students, with the WoS search terms TS = (“university student*” OR “college student*” OR “higher education” OR “undergrad student*” OR “master’s student*” OR “postsecondary education” OR “undergraduate*” OR “tertiary education” OR “postsecondary education” OR “doctoral student*” OR “Ph.D. student*”). Thirdly, to focus our research topic on internet addiction, the search terms are set as follows: TS = (“online addiction” OR “internet addiction” OR “internet disorder” OR “network addiction” OR “internet addiction” OR “net addiction”).

### 2.2 Data screening and extraction

**Figure 1:**
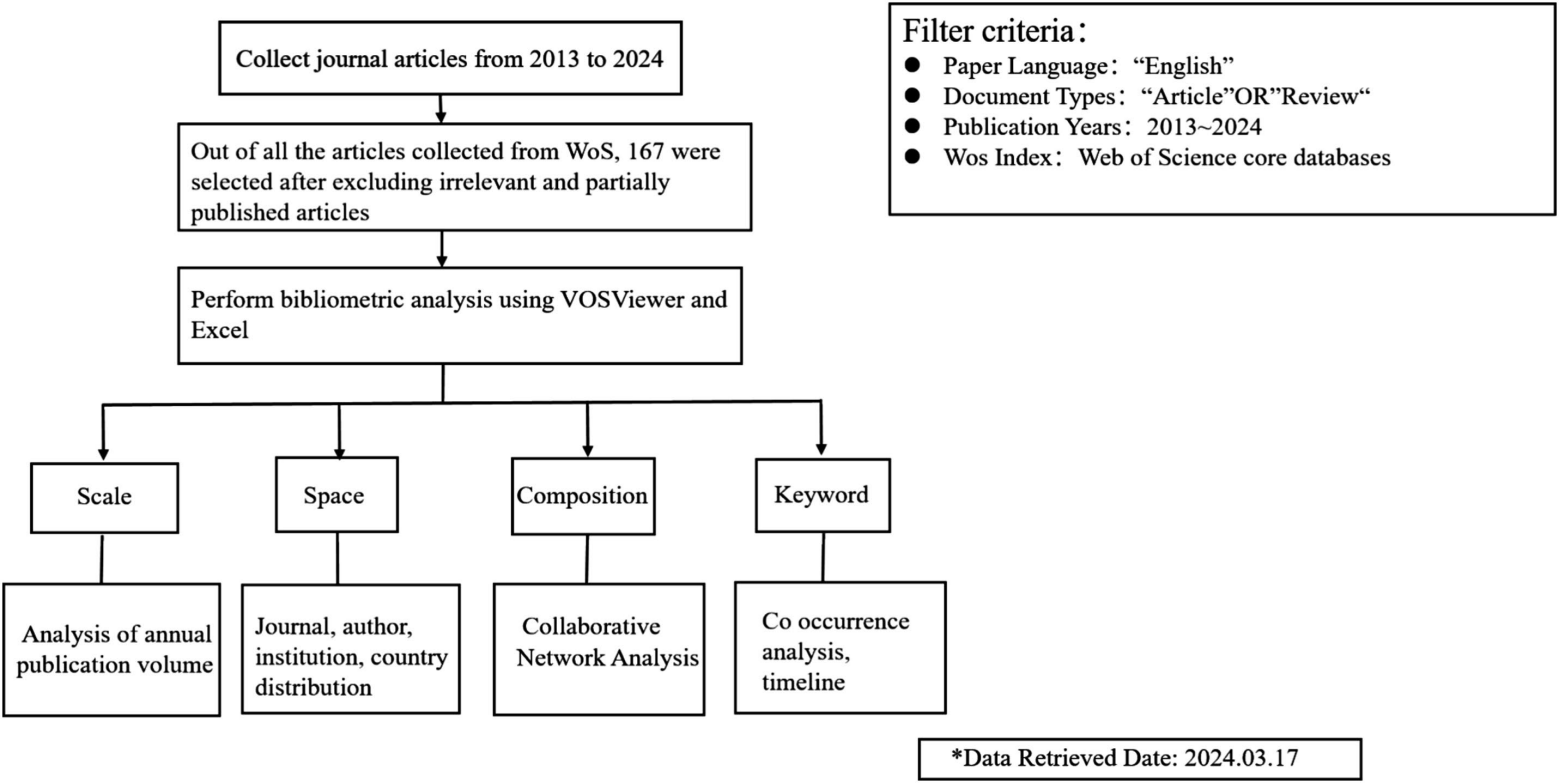
The flowchart of data collection and filtering.

The inclusion criteria specified a time span from 2013 to 2024, with literature types limited to “Article” and “Review,” and the language restricted to English. Additionally, during the data processing, retrieval, and selection phases, two reviewers independently and sequentially screened potential studies based on predefined inclusion and exclusion criteria. Differences in judgments were resolved through discussion, and consensus was reached. Following screening of titles, abstracts, and full-text articles, a total of 302 articles were initially identified in the WoS database, from which 167 articles met the final inclusion criteria. Article retrieval and data extraction were conducted on March 1, 2024, within a single day, to mitigate potential bias resulting from daily database updates. The data collection process and analysis methods are depicted in Figure 1.

### 2.3 Data analysis tools and methods

VOSviewer (Version: 1.6.20, Netherlands) is an open-source software designed for viewing and constructing bibliometric maps, which was developed by the Science and Technology Research Center of Leiden University and released on October 31, 2023. In VOSviewer, the analysis units include journals, publications, citations, authors, or countries. The nodes in the map represent units in the analysis, with the size of the nodes indicating their proportional importance and their position reflecting the similarity between different nodes in the map. The lines connecting different nodes represent the relationships between nodes, with the thickness of the lines indicating the strength of these relationships, and the color of the nodes representing different clusters, which are delineated based on the degree of association between nodes (Van Eck and Waltman, 2014). The construction of the network map follows a distance-based method, comprising three steps. Firstly, the software standardizes each node sequentially to eliminate differences between nodes. Secondly, it constructs a two-dimensional map where the distance between nodes reflects the similarity between them. Thirdly, VOSviewer categorizes closely related nodes into the same cluster to achieve clustering goals (Van Eck et al., 2010).

In this analysis, bibliographic data was exported from the WoS database in Plain Text file format and subsequently imported into VOSviewer using the “Create a map based on bibliographic data” module. First, we conducted a co-authorship analysis, selecting “Authors” and “Countries” as the units of analysis, with a “Minimum number of documents” threshold of 1 and 2, respectively. Next, we performed a co-citation analysis, using “Cited sources” as the unit of analysis, with a “Minimum number of citations of a source” threshold of 20. Finally, we conducted a co-occurrence analysis, selecting “Author keywords” as the unit of analysis, with a “Minimum number of occurrences of a keyword” threshold of 7.

### 2.4 Language translation and polishing

In this study, we employed ChatGPT-3.5 (OpenAI) to perform Chinese-English translation of the manuscript with subsequent English polishing to enhance readability and all AI-generated content underwent rigorous review and edition by authors to ensure compliance with academic standards.

## 3 Results

### 3.1 Overall development trajectory

**Figure 2:**
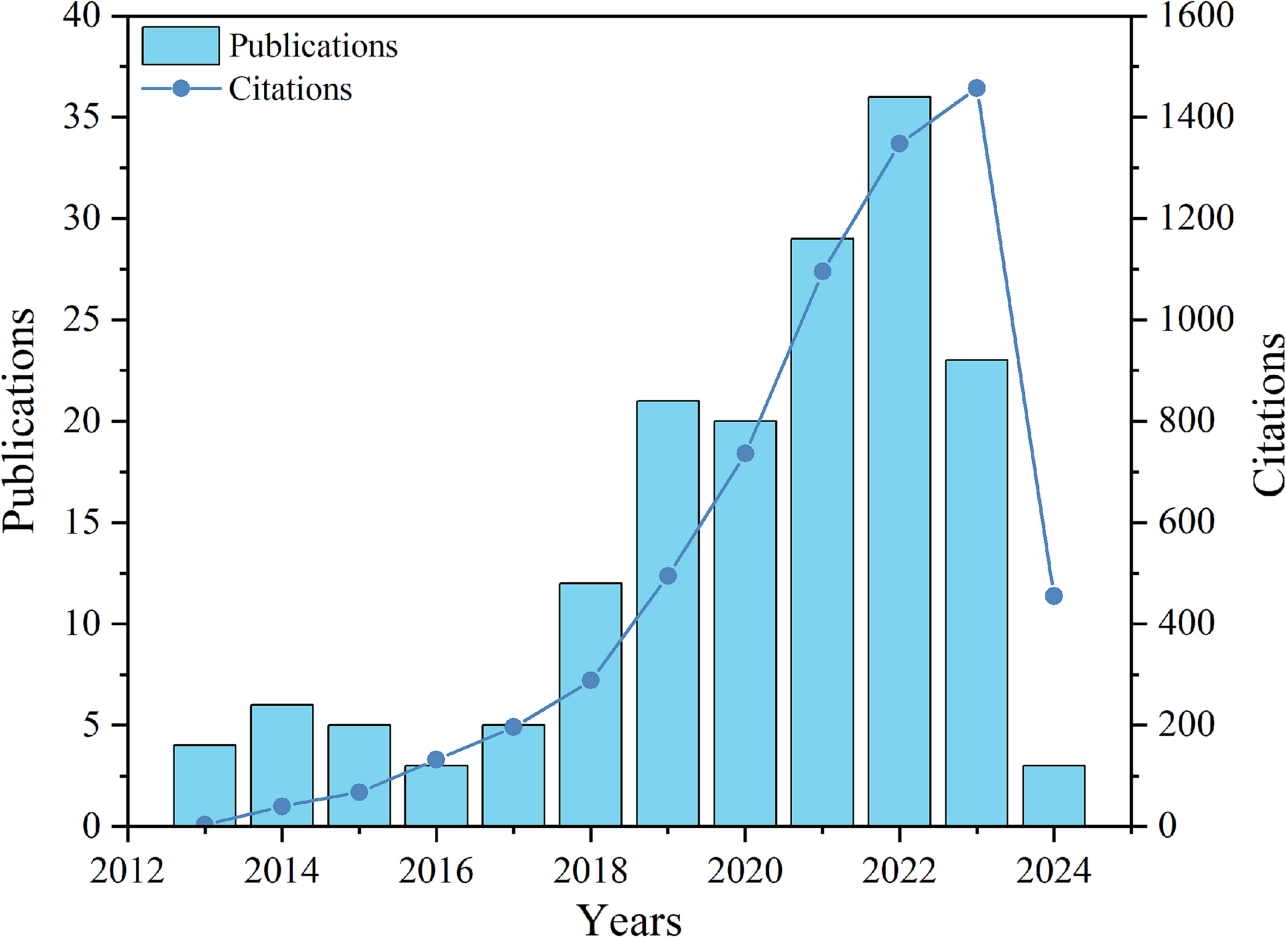
The publications and citations per year in the field of social media dependence and internet addiction among college students.

The annual change in the number of publications per year provides a clear illustration of the development pattern in a field in recent years. Figure 2 shows the development trajectory of publications and citations from 2013 to 2024 regarding social media dependence and college students’ online addiction. Generally, over the past 2013–2024 period, there has been a growing interest among scholars in the issue of college student internet disorder, and the research can be roughly divided into three stages: The first stage, the preliminary exploration stage (2013–2017), where a small number of publications and citations were found in the WoS database. This may be internet penetration was low, and cyber addiction was not a widespread issue at that time (Lozano-Blasco et al., 2022). The second stage, the take-off stage (2018–2022), where there was a significant increase in the number of publications and citations in the WoS database, especially in 2022, where the number of publications was almost double that of 2020. This could be due to the growing popularity of the internet, the rise of social media, and a surge in the number of users (Kaplan and Haenlein, 2010). Additionally, since 2019, the global outbreak of COVID-19 has caused people to quarantine at home. The use of social media has emerged as an effective means of alleviating anxiety, potentially leading to a notable increase in issues related to internet addiction. The third stage is the stabilization stage (2023–2024). During this period, although the number of publications and citations in the database decreased, it still remained at a relatively high level. The trajectory of citation counts was similar to that of the publications data. This might be due to, with the end of the pandemic, the demand for college students to use social media decreased, resulting in the number of literatures basically returning to the level before the pandemic (Goudeau et al., 2021).

### 3.2 Dominant productivity

#### 3.2.1 Main publication sources

**Figure 3:**
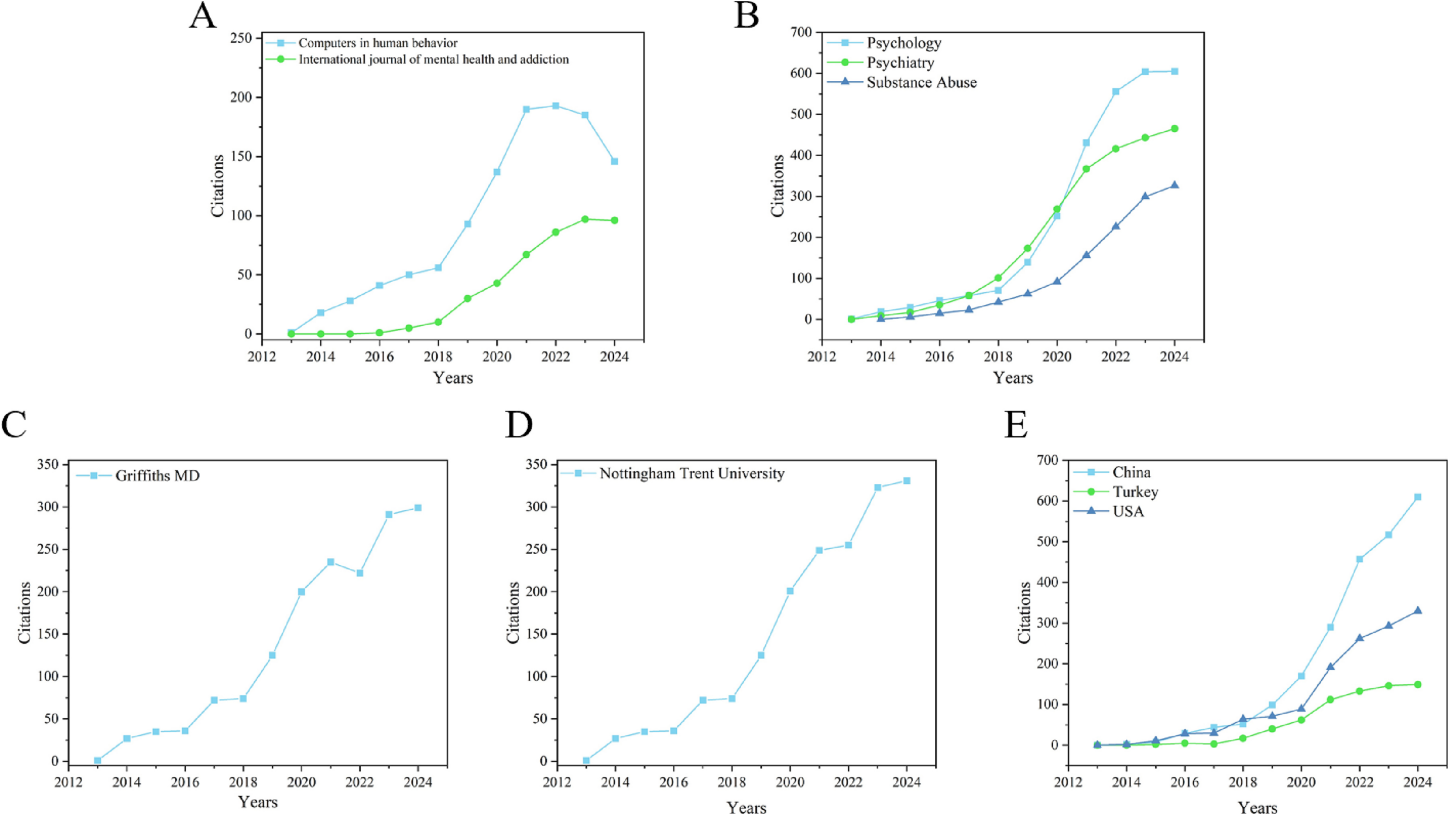
Annual citations from 2013 to 2024 in the field of social media dependence and internet addiction among college students. **(A)** Top 2 publication sources. **(B)** Top 3 research fields. **(C)** Top 1 author. **(D)** Top 1 institution. **(E)** Top 3 countries.

Overall, 167 articles were published in 96 journals in the WoS dataset. Table 1 lists the top 10 sources of publications. It is evident from the table that the journals *Computers in Human Behavior* and “*International Journal of Mental Health and Addiction*” are the most productive publication sources in the field, each accounting for 6.587% of the WoS database. *Computers in Human Behavior* focuses on the psychological aspects of computer use, with an average citation count of 90.27. *International Journal of Mental Health and Addiction* provides the latest information on research, policy, phenomenology, literature, and treatment related to mental health and addiction, with an average citation count of 31. Next is *Addictive Behaviors*, accounting for 4.192% of the WoS database, and *Addicta the Turkish Journal on Addictions*, accounting for 3.593%. As seen from Table 1, existing articles are mainly published in journals related to computer behavior, psychology, addictive behaviors, psychiatry, and public, environmental, and occupational health, with the largest number of publications in computer behavior and psychology, as well as addictive behaviors. Additionally, to understand how these articles were cited over time, we counted the annual citations of the top 2 publication sources, as shown in Figure 3A.

**Table 1:** Top 10 publication sources distributed by publications.

Rank	Source title	Publication count (%)	Citations	Average citation	IF(2022)	JCR(2022)
1	Computers in human behavior	11 (6.587)	993	90.27	9.9	Q1
1	International journal of mental health and addiction	11 (6.587)	341	31	8	Q1
3	Addictive behaviors	7 (4.192)	317	45.29	5	Q1
4	Addicta the Turkish journal on addictions	6 (3.593)	43	7.17	0.9	Q4
4	Journal of behavioral addictions	6 (3.593)	692	115.33	7.8	Q1
4	Telematics and informatics	6 (3.593)	589	98.17	8.5	Q1
7	Current psychology	4 (2.395)	64	16	2.8	Q2
7	International journal of environmental research and public health	4 (2.395)	157	39.25	–	Q2
7	PLoS One	4 (2.395)	319	79.75	3.7	Q2
10	Acta psychologica	3 (1.796)	3	1	1.8	Q3

#### 3.2.2 Main research fields

Table 2 shows the top 10 fields contributing to the study of the impact of social media on college student internet addiction. More than half of the records in the WoS dataset were published in the fields of psychology (40.719%) and psychiatry (26.347%). These two fields are the main research areas for studying college students’ online addiction, with significant achievements in analyzing and researching the causes and development of digital addiction at the psychological and psychiatric levels. The field of substance abuse, related to psychiatry, also showed significant influence in this study, publishing 26 studies, accounting for 15.569% of the publications. Next is the field of public, environmental, and occupational health, which published 11 papers, accounting for 6.587%. Other related research is mainly concentrated in fields such as computer science, other topics in science and technology, education and educational research, information science and library science, environmental and ecological science, and other topics in social sciences. Additionally, the annual citations of the top 3 research fields are shown in Figure 3B.

**Table 2:** Top 10 research fields distributed by publications.

Rank	Research areas	Publications, *n*(%)	Citations	Average citation	H-index
1	Psychology	68 (40.719)	2,232	32.82	25
2	Psychiatry	44 (26.347)	1,942	44.14	18
3	Substance Abuse	26 (15.569)	974	37.46	14
4	Public Environmental Occupational Health	11 (6.587)	252	22.91	7
5	Computer Science	9 (5.389)	410	45.56	7
5	Science Technology Other Topics	9 (5.389)	348	38.67	5
7	Education Educational Research	8 (4.79)	31	3.88	3
7	Information Science Library Science	8 (4.79)	775	96.88	8
9	Environmental Sciences Ecology	6 (3.593)	178	29.67	5
9	Social Sciences Other Topics	6 (3.593)	262	43.67	5

#### 3.2.3 Main authors, institutions, and countries/regions

Table 3 lists the researchers who have published the most papers in the field from 2013 to 2024. Griffiths Md (17) is undoubtedly the leading researcher, hailing from the institution Nottingham Trent University that has published the most papers, accounting for 10.18% of the WoS database and ranking first in contributions to the field. His annual citations of are shown in Figure 3C. Next is Lin CY (4), ranking second with 2.395% of the WoS database, who has also made significant contributions to the field. There are also seven authors, including Andreassen CS (3), Foroughi B (3), Iranmanesh M (3) and so on.

**Table 3:** Leading authors distributed by publications.

Authors	Publication count, *n*(%)	Citations	Average citation	H-index
Griffiths MD	17 (10.18)	1,341	78.88	13
Lin CY	4 (2.395)	187	46.75	3
Andreassen CS	3 (1.796)	811	270.33	3
Foroughi B	3 (1.796)	116	38.67	3
Iranmanesh M	3 (1.796)	116	38.67	3
Kuss DJ	3 (1.796)	367	122.33	3
Pakpour AH	3 (1.796)	186	62	3
Pallesen S	3 (1.796)	811	270.33	3
Zhang Y	3 (1.796)	127	42.33	2
Affouneh S	2 (1.198)	49	24.5	2
Berte DZ	2 (1.198)	49	24.5	2
Brailovskaia J	2 (1.198)	166	83	2
Chiu SL	2 (1.198)	215	107.5	2
Cricenti C	2 (1.198)	8	4	1
Elphinston RA	2 (1.198)	17	8.5	1
Fraschetti A	2 (1.198)	8	4	1
Giannini AM	2 (1.198)	8	4	1
Gullo MJ	2 (1.198)	17	8.5	1
Garcés-delgado Y	2 (1.198)	11	5.5	1
Hong FY	2 (1.198)	215	107.5	2

Table 4 lists the leading institutions in terms of publication output in this field. Nottingham Trent University (WoS = 19) ranks first in this area, being the most influential and prolific educational institution in terms of publishing outputs, and its annual citations of are shown in Figure 3D. Following are Monash University (WoS = 4) from Australia, Universiti Sains Malaysia (WoS = 4) from Malaysia, and the University of Bergen (WoS = 4) from Norway, each contributing the same amount to the field despite their different geographical locations. Additionally, institutions from China, such as I-Shou University (WoS = 4), the Chinese Academy of Sciences (WoS = 4), and the University of Science and Technology of China CAS (WoS = 4), have also made significant contributions. It can be observed from the table that, with the exception of Nottingham Trent University, the publication outputs of most institutions in this field are almost identical.

**Table 4:** Leading institutions distributed by publications.

Institution	Publication count, *n*(%)	Citations	Average citations	H-index
Nottingham Trent University	19 (11.377)	1,423	74.89	14
Chinese Academy of Sciences	4 (2.395)	113	28.25	3
I-Shou University	4 (2.395)	304	76	4
Monash University	4 (2.395)	79	19.75	3
Universiti Sains Malaysia	4 (2.395)	141	35.25	4
University of Bergen	4 (2.395)	858	214.5	4
University of Science Technology of China Cas	4 (2.395)	113	28.25	3
Beijing Normal University	3 (1.796)	19	6.33	2
Edith Cowan University	3 (1.796)	116	38.67	3
Jonkoping University	3 (1.796)	186	62	3
Kermanshah University of Medical Sciences	3 (1.796)	111	37	2
National Cheng Kung University	3 (1.796)	186	62	3
National Cheng Kung University Hospital	3 (1.796)	186	62	3
Necmettin Erbakan University	3 (1.796)	1	0.33	1
Ruhr University Bochum	3 (1.796)	179	59.67	3
Sakarya University	3 (1.796)	16	5.33	1
Sapienza University Rome	3 (1.796)	26	8.67	2
Universiti Malaya	3 (1.796)	188	62.67	3
Xi'an Jiaotong University	3 (1.796)	131	43.67	3

Table 5 lists the leading countries/territories in studying the impact of social media on college student cyber addiction. China is undoubtedly in the leading position, having published 39 articles (23.353%) on the WoS database, ranking first. Turkey follows with 26 publications (15.569%), and the United States is in third place with 24 publications (14.371%) on the WoS database. Their annual citations can be seen in Figure 3E. Additionally, the top 10 countries/territories in the WoS dataset are primarily distributed in Asia (Malaysia, Iran, and South Korea), Oceania (Australia), and Europe (England, Spain, and Italy). This indicates that research on the impact of social media on college student internet disorder has received varying degrees of attention worldwide, particularly in Asia and Europe.

**Table 5:** Leading countries/territories distributed by publications.

Countries/territories	Publication count, *n*(%)	Citations	Average citation	H-index
China	39 (23.353)	948	24.31	16
Turkey	26 (15.569)	571	21.96	11
USA	24 (14.371)	1,066	44.42	13
England	23 (13.772)	1,449	63	15
Spain	13 (7.784)	225	17.31	6
Australia	10 (5.988)	296	29.6	7
Italy	9 (5.389)	289	32.11	7
Malaysia	9 (5.389)	337	37.44	7
Iran	8 (4.79)	318	39.75	6
South Korea	8 (4.79)	169	21.13	6

### 3.3 Scientific collaboration network analysis

**Figure 4:**
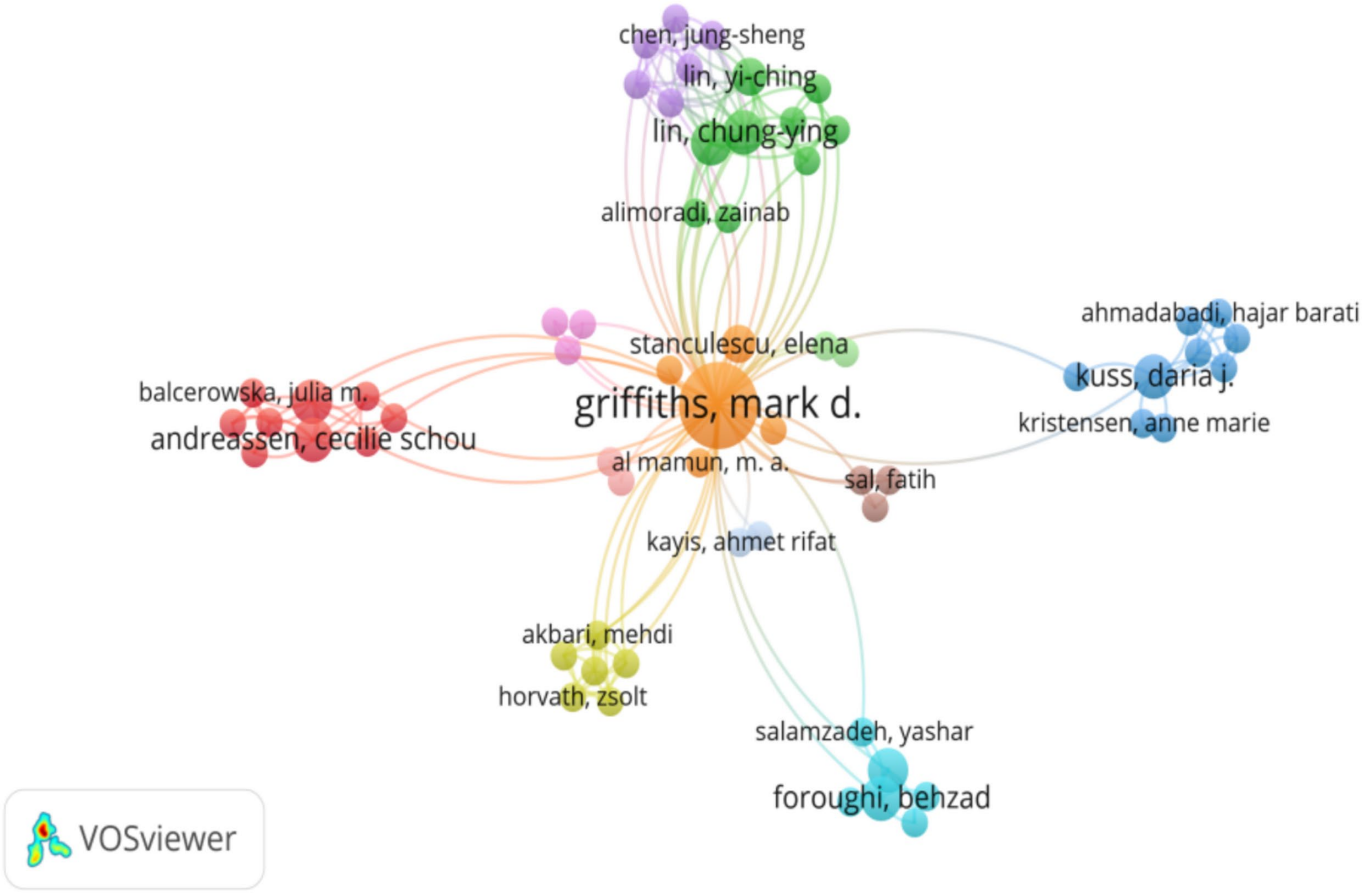
Scientific collaboration networks between authors. Only researchers with one or more publications were considered in the analysis (*n* = 622).

In this study, we utilized VOSviewer to delve into the potential scientific collaboration networks within the field of the impact of social media on college student web addiction over the past decade, encompassing researchers and countries/territories. Figure 4 illustrates the network of collaboration among authors within the dataset. In the VOSviewer software, we set the size of the nodes to be proportional to the number of posts by the author, in order to measure the importance of the author in the field. The thickness of the connection between nodes indicates the strength of cooperation between authors, which is determined by the number of co-posts. The greater the number of collaborations, the thicker the connection. Cluster analysis uses the software’s default similarity-based clustering algorithm. The similarity threshold is appropriately adjusted according to the recommended values of the software and the characteristics of the data in this study to ensure that the clustering results can clearly show the different research groups and cooperation patterns. All researchers who have published at least one article were considered in this analysis, resulting in the inclusion of 622 researchers in the network construction. The map shows the existence of multiple productive collaboration networks within the field, contributing to its advancement. Centrally, there are 12 cross-territorial collaboration networks, characterized by close connections and a significant number of researchers. Among these, the largest collaboration network (red cluster) is formed by four researchers from the University of Gdansk and five researchers from the University of Bergen. The second-largest collaboration network (green cluster) is composed of three researchers from Qazvin University of Medical Sciences (QUMS) and six researchers from China. Additionally, the blue galaxy cluster consists of six higher education research institutions from different countries such as the United Kingdom and Iran, while the yellow galaxy cluster includes two researchers from Hungary and four researchers from Iran, with close collaboration with the orange galaxy cluster.

**Figure 5:**
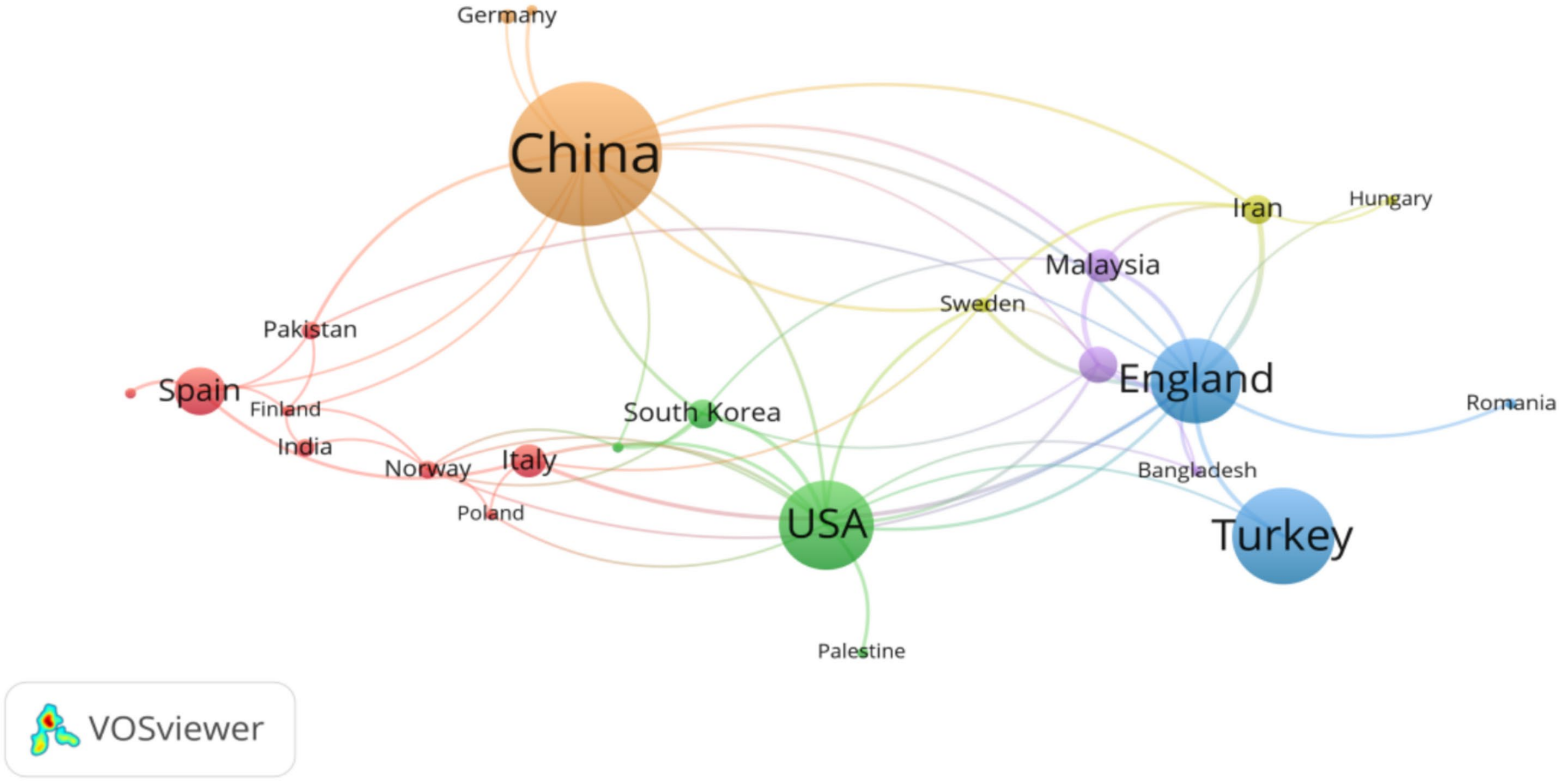
Scientific collaboration networks between country/territories. Only countries with 2 or more publications were considered in the analysis (*n* = 28).

The transnational collaboration network for studying the impact of social media on college student internet disorder over the past decade is depicted in Figure 5. In this analysis, only research collaborations between countries that have published two or more papers were considered, with 28 out of 51 countries being included in the network analysis. For the setting of nodes, the size of the national node represents the frequency of its appearance in research. The more occurrences, the larger the node, which means that the country has a higher active level of research in this field. The thickness of the connection between nodes indicates the degree of cooperation between countries. This is measured by the number of papers jointly published by the two countries. The more papers jointly published, the thicker the connection. China, as represented by the largest node in the map, signifies the highest frequency of appearance in the field of research and the formation of robust partnerships with other countries/territories (brown cluster). Overall, a certain scale of international cooperation groups have been formed in this field, with the largest cluster (red cluster) comprising two Asian countries (India and Pakistan) and six European countries (Finland, Italy, Norway, Poland, Portugal, and Spain).

### 3.4 Journal co-citation analysis

To explore the disciplines underlying the foundations of research on social media dependence and internet addiction among college students, we conducted a journal co-citation analysis using VOSviewer. The strength of co-citation relationships is measured by co-citation frequency. Two journals are considered co-cited when they are both cited by a third journal. The more frequently a pair of journals is cited together by other journals, the stronger their co-citation relationship. Journals that are frequently co-cited are considered to share a common theoretical and semantic foundation (Hernandez-Torrano et al., 2020). Therefore, in our study, clusters of frequently co-cited journals can be interpreted as the disciplinary foundation of research on social media dependence and internet addiction among college students. In this analysis, only journals that have been cited at least 20 times were considered, with 71 out of a total of 3,419 journals being included in the analysis. The nodes in the map represent journals, with their size reflecting the number of co-citations with other journals. The color represents journal clusters, with journals within the cluster having higher co-citation relationships and stronger disciplinary connections.

**Figure 6:**
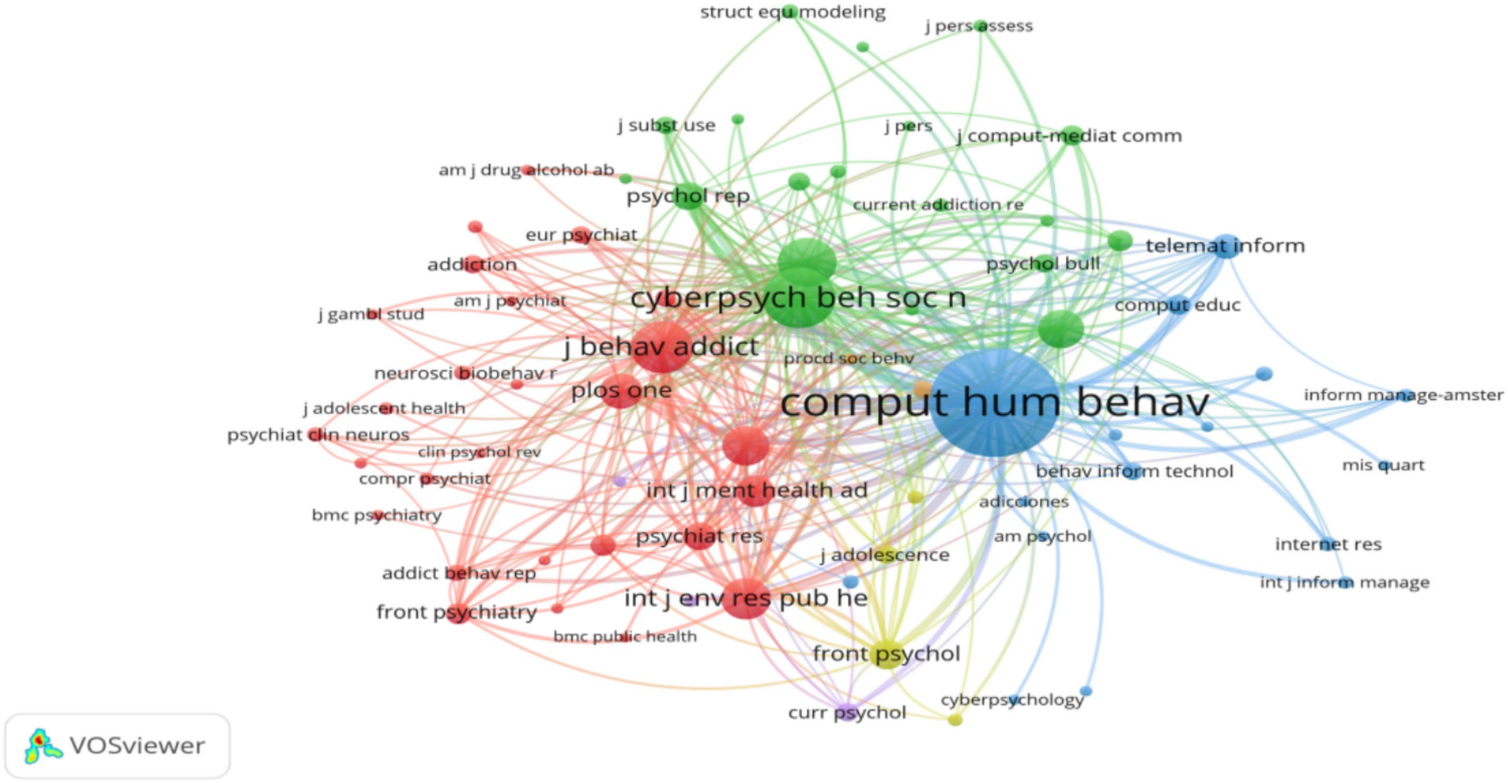
Visualization network of the co-citation in journals. Only citations with 20 or more citations were considered in the analysis (*n* = 71).

From Figure 6, the largest node, *Computers in Human Behavior*, belongs to the blue cluster, which also includes journals such as *Addiction Research and Theory*, *American Psychologist*, *Behavior and Information Technology*, “*Internet Research-Electronic Networking Applications and Policy* and *New Media and Society*. This group can be characterized as a scientific cluster related to psychology, addictive behavior research, internet research, and new media. Overall, this analysis showed that the research field on the impact of social media on college student internet addiction exhibits a degree of interdisciplinary nature. For instance, on the left side of the map, the red cluster represented by *Journal of Behavioral Addictions* includes journals related to addictive behavior, psychiatry, public health, environmental science, and sociology. At the top, the green cluster represented by *Cyberpsychology Behavior and Social Networking* includes journals related to education, sociology, psychology, and drug abuse that contribute to the research and development of the field. In the corner, the yellow cluster represented by *Frontiers in Psychology* includes journals related to adolescence, family, psychological development, and puberty. There is also a purple cluster that includes journals related to contemporary psychology, psychiatry, and addictive behavior. The co-citation journal analysis indicates that research on the impact of social media dependence on college student web addiction is, to some extent, interdisciplinary, encompassing fields such as behavioral addiction, psychology, psychiatry, sociology, and computer science.

### 3.5 Visual analysis of research trends and hotspots

**Figure 7:**
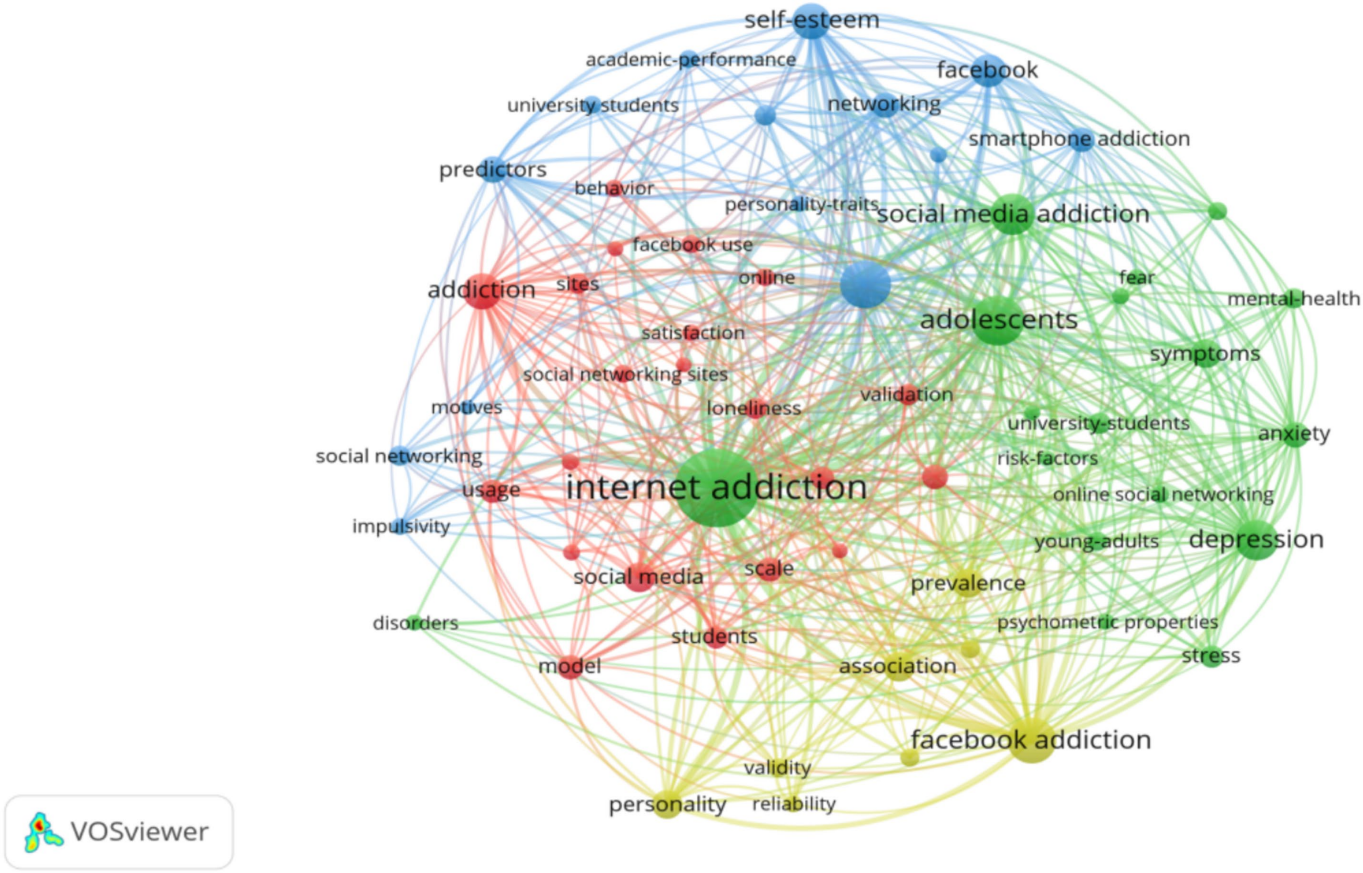
Visualization network of keyword co-occurrence analysis. Only keywords with 7 or more occurrences were considered in the analysis (*n* = 61).

From January 2013 to March 2024, the research hotspots in the field related to the impact of social media on college student digital disorder are visualized in Figure 7. Co-occurrence analysis of keywords from all publications in the dataset provides a visual representation. Co-occurrence rate refers to the frequency of two thematic terms appearing in the same article, aiming to indicate the relationship between the terms. In this analysis, we considered only keywords that appeared 7 times or more, with 61 out of 813 keywords being included in the analysis. Keywords represent the core themes of articles, and their frequency reflects the level of research interest in the topics. Keywords with higher frequencies are represented by larger nodes, and the thickness of the lines represents the strength of co-occurrence between different keywords. In the graph, the size of a keyword node is proportional to the frequency of the keyword. The higher the frequency, the larger the node, indicating that the keyword has received higher attention in the research. The thickness of the connection between nodes indicates the strength of keyword co-occurrence, that is, the more times two keywords appear at the same time in the same article, the thicker the connection, the closer the correlation between them.

The keywords with the highest frequency in the dataset include “Facebook addiction” (frequency: 51), “social media addiction” (frequency: 43), “depression” (frequency: 41), “self-esteem” (frequency: 33), “association” (frequency: 24), “personality” (frequency: 23), “prevalence” (frequency: 23), “predictors” (frequency: 20), “anxiety” (frequency: 19), “networking” (frequency: 18), “usage” (frequency: 16), “loneliness” (frequency: 13), and “life satisfaction” (frequency: 12). This indicates that in the context of net addiction among college students in higher education systems, social media addiction constitutes a significant proportion, and social media has a severe negative impact on college students’ cyber addiction. The mechanism of this impact is closely related to factors such as the rapid development of the internet, the high penetration of social media, the lack of real-life interpersonal interactions, the explosion of online information, and individual personality differences, representing relevant phenomena in the research field. Most studies are related to specific social media platforms such as Facebook and Twitter, as well as the time investment of college students in using social media and the mental health statuses of social media-addicted college students, including social pressure, self-identity, anxiety, and other psychological health issues. Cluster analysis results showed that these four themes may constitute the research hotspots in the field of social media and college student internet addiction over the past decade. Firstly, researchers generally show interest in the mediating factors between social media and college student online disorder, with high-frequency keywords such as “Facebook use,” “gender differences,” “mental health status,” “internet,” “social networking frequency,” “social media site size,” “number of online users,” and “evaluation of social media addiction models” (red cluster) frequently appearing in the map. Secondly, the mental health challenges faced by college students and the issues with social media use are another widely discussed topic, with keywords including “anxiety,” “depression,” “fear of missing out,” “stress,” and “social media risks” (green cluster). The third popular area is the study of college students’ motivations for using social networks, with keywords such as “college student motivation,” “impulsivity,” “life satisfaction,” “social personality traits,” and “prediction” (blue cluster).

**Figure 8:**
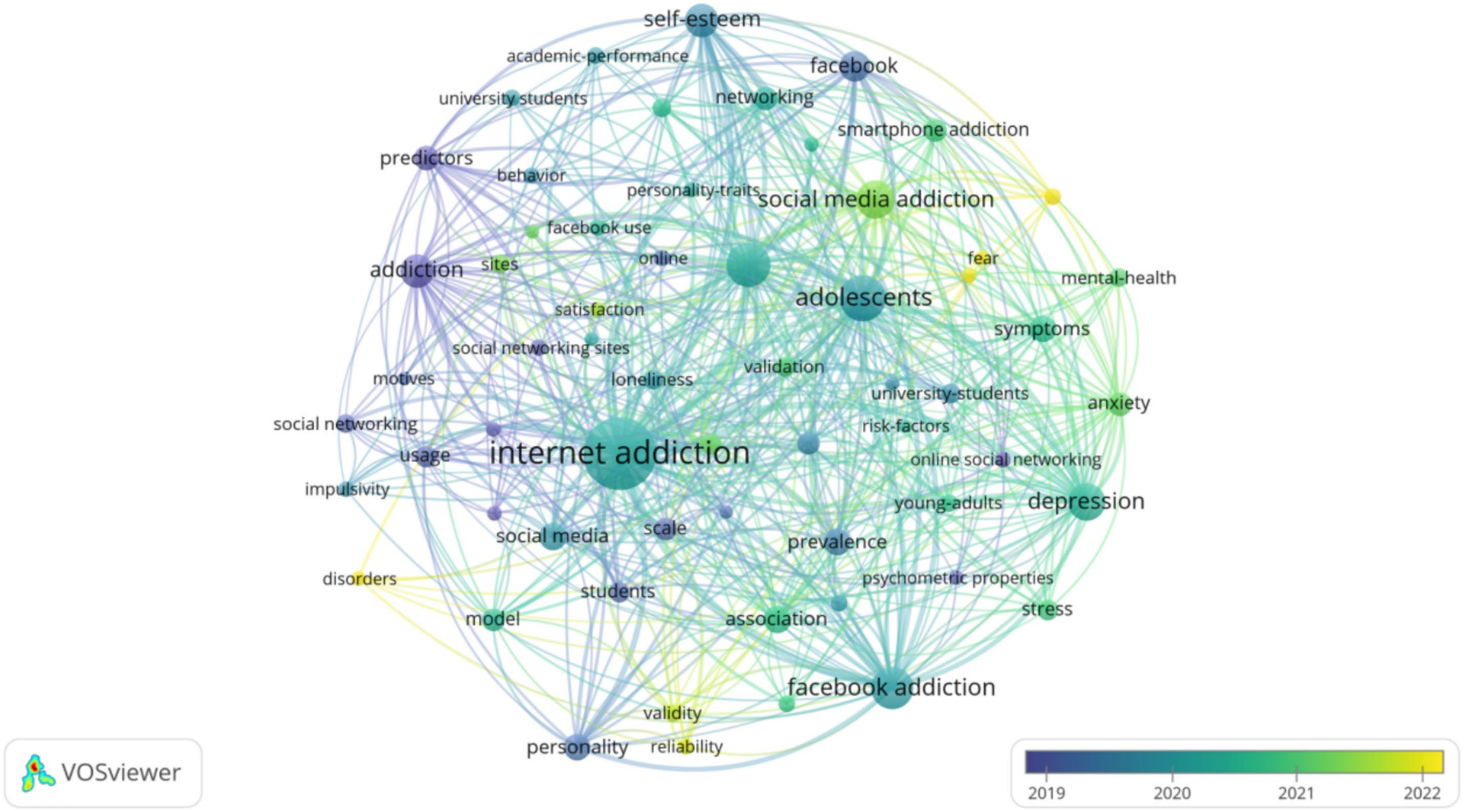
Visualization network of keywords average year map from 2012 to 2024.

Additionally, to illustrate the trend of research topics in the field from 2013 to 2024, a temporal visualization analysis of keywords was conducted, with the color legend representing the average year of high-frequency keyword appearance (Li and Liu, 2020), as shown in Figure 8. Deep blue represents the average year of the earliest appearance of the keyword, and yellow represents the average year of the latest appearance. The size of the nodes represents the popularity of the research, and the legend indicates the average year. Around 2019, the research hotspots mainly focused on the popularity of social networking sites and the motivations behind college students’ social media addiction behaviors. The research topics of greatest interest were centered on the impact of mental health status from 2020 to 2021. By around 2022, the specific analysis of the diversification of social networking sites, the dependence of college students on social media, and the impact of personality trait differences on social media’s influence on college student online addiction also piqued researchers’ interest, and research on the potential mechanisms of college student internet addiction gradually increased.

## 4 Discussion

Overall, these results indicate that in the fields of social media dependence and internet addiction among college students, the number of literatures has experienced growth and decline, which is closely related to the development of the internet and the epidemic. Core contributors such as Nottingham Trent University and Griffith University in Maryland led this research, and scientific researchers from China also occupied an important position in this field. The co-collaboration network analysis shows that although there are collaboration networks between authors and countries, their scales are limited and there is still room for expansion. The co-citation analysis of the journals point out that the interdisciplinary integration in this field is obvious, mainly involving behavioral addiction, psychology, sociology, and computer science. The analysis of keywords shows that the popular research hotspots in the fields of social media dependence and Internet addiction among college students mainly focus on exploring the relationship between social media dependence and online addiction among college students and the mediating factors affecting online addiction among college students.

Therefore, future research must strengthen the cooperation between developed countries and other countries, as well as between institutions. Meanwhile, it is necessary to promote cooperation among researchers from different disciplines, which will lead to higher universality and quality of research in this field and promote the comprehensive development of this field. Moreover, in the keyword co-occurrence analysis, several high-frequency keywords have attracted our attention. On one hand, terms such as “Facebook addiction” (frequency: 51), “social media addiction” (frequency: 43), and “association” (frequency: 24), indicate that the relationship between social media dependence and internet addiction is a widely discussed and central theme within the field. On the other hand, keywords like “depression” (frequency: 41), “self-esteem” (frequency: 33), and “anxiety” (frequency: 19), reflect another significant research focus— the mediating factors influencing social media dependence and internet addiction among college students. This thematic mapping aligns with the centrality metrics from the network analysis, where these factors emerge as pivotal nodes in the map, which will be discussed in detail in the subsequent parts. Recently, given an increasing scholars have focused on the research of the mechanisms of social media dependence and cyber addiction among college students and related intervention strategies, it is necessary for us to systematically sort out the past research results, so as to improve the cognitive system of the problems of social media dependence and cyber addiction among college students and provide theoretical support for solving this phenomenon.

### 4.1 The relationship between social media and college student internet addiction

The current situation of internet addiction is severe and has garnered widespread attention from various sectors of society (Rutter, 1987). College students are the largest potential group of internet addicts, exhibiting the most severe symptoms (Li et al., 2021). Surveys indicate a high detection rate of online addiction among college students (Al Shawi et al., 2021), with an average global prevalence of approximately 6.0%. A comprehensive incidence rate of internet disorder among Chinese college students is as high as 11%, European countries college students at 28% and the Middle East is 10.9%, with this ratio showing an upward trend (Cheng et al., 2021), making cyber addiction a concerning global disease.

Excessive use of social media can lead to severe technology addiction behaviors. Compared to traditional media such as television, newspapers, and magazines, social media platforms attract users due to their unique features of interactivity, anonymity, openness, and virtuality. The large volume of information, rich stimuli, and rapid update speed of social media platforms greatly cater to the psychological characteristics of college students, such as curiosity, exploration, and a desire for freedom. The impact of social media dependence on college student technology disorder cannot be ignored (Shao et al., 2018; D’Arienzo et al., 2019).

Research indicates that net addiction has a particularly significant impact on college students, as they are still undergoing psychological and social development (Shen et al., 2020; Baturay and Toker, 2019). Reports suggest that net addiction psychologically lowers college students’ self-esteem, self-confidence, social self-efficacy, and academic self-efficacy, and triggers feelings of loneliness. High participation in social networking sites (SNS) among college students is associated with adverse psychological outcomes such as depression, anxiety, and loneliness (Bottaro et al., 2024; Primack et al., 2017), and it can interfere with sleep and hinder academic success (Woods and Scott, 2016), even leading to severe economic stress. Physically, it can cause eye fatigue, vision loss, physical weakness, muscle soreness, tension, anxiety, headaches, anorexia or appetite loss, and in the long term, it can induce cardiovascular diseases, gastrointestinal neurosis, tension headaches, and in severe cases, mental confusion, stupor, or even sudden death. Furthermore, scholars have found through survey questionnaires that online addiction and social media addiction significantly affect adolescents’ social connections (Savci and Aysan, 2017), and college students are in a critical stage of life, forming habits that may persist into old age (Arnett, 2000). Identifying and addressing addiction patterns during this period can avoid future impacts (Kuss et al., 2014). As a dominant group of social network users, understanding students’ addiction status can help in developing tailored intervention strategies. Therefore, we must recognize the importance of social media leading to internet addiction, pay attention to the potential impact of social media on individual mental health, ensure healthy social media use, understand the roots of social network addiction, and help alleviate these problems, ensuring healthy social media use.

### 4.2 Mechanisms of internet addiction in college students related to social media dependence

#### 4.2.1 Behavioral explanation

From a behavioral perspective, over time, various types of social media platforms have become ubiquitous (Lee, 2010). Using social media is one of the most common activities among children and adolescents today, providing channels for entertainment and communication. In recent years, the number of young users of social media has grown exponentially (Gabarron and Wynn, 2016). According to Gitnux’s report, almost all American college students (about 98%) use social media, with 50% of students spending 1–5 h on social media every day (Dwivedi et al., 2023). There have also been studies exploring the use of social media in the business and consumer market, with Facebook as an example to describe how different content on social media affects consumers’ perceptions of brands (Hajli, 2014). Furthermore, research has focused on the impact of social media itself, including on business strategies (Lee, 2010), consumers (Jones et al., 2015), companies (Richards et al., 2015), teenagers (Erdoğmuş and Cicek, 2012), brand loyalty (Karakiza, 2015), and the public sector (Alhassan et al., 2018). This demonstrates that social media has permeated various aspects of people’s lives, and using social media has become a behavioral habit in daily life. Studies have reported that social media has had adverse effects, such as relationship issues and symptoms of cyber addiction (Cheng et al., 2021). Excessive time spent on social media and low self-control are more likely to lead to negative outcomes and increase the incidence of internet disorder.

#### 4.2.2 Physiological explanation

From a physiological perspective, research has found that addictive behaviors are associated with six different neurotransmitters, including gamma-aminobutyric acid (GABA), acetylcholine, norepinephrine, dopamine, serotonin, and beta-endorphin. Currently, research on norepinephrine and dopamine is more prevalent. Norepinephrine primarily regulates emotions and maintains sleep states (Márquez et al., 2017); dopamine plays a role in balancing acetylcholine, stimulating the pleasure center, regulating emotions, and influencing cognitive processes, as well as inhibiting prolactin secretion. Studies have shown that prolonged use of social media can keep the brain’s central nervous system in a state of high excitement, leading to abnormal increases in adrenaline levels, excessive sympathetic nerve excitement, and elevated blood pressure. It can also increase dopamine levels in the brain, a substance similar to adrenaline that can cause heightened excitement in the short term. The recovery of dopamine neurotransmitters can eliminate anxiety, re-experience pleasure, and then lead to the occurrence of internet addiction behaviors (Everitt and Robbins, 2005).

#### 4.2.3 Psychological explanations

##### 4.2.3.1 ACE model

Young proposed the ACE model, which posits that anonymity, convenience, and escapism are three key characteristics of the internet that contribute to net addiction. Anonymity refers to the ability to change one’s name and gender online without revealing one’s real identity. Convenience refers to the ease of online shopping, communication, and socializing. Escapism refers to the tendency to vent one’s emotions online when faced with difficulties in real life. For example, research has shown that certain parenting styles, including warmth, attachment, and responsiveness, are associated with fewer digital addiction behaviors (Kim and Kim, 2015; Zhang et al., 2019), while dysfunctional parent–child relationships lead to students using the internet extensively to escape (Shek et al., 2018; Sun and Wilkinson, 2020; Zhao et al., 2020).

##### 4.2.3.2 Cognitive-behavioral model

The cognitive-behavioral model, proposed by Davis (2001), focuses on maladaptive cognitions and the individual’s vulnerability. It suggests that maladaptive cognitions, along with an individual’s vulnerability and significant life events, are the necessary and sufficient conditions for the development of internet disorder. When individuals experience depression, anxiety, and are under stress, they are more likely to become addicted to the internet. This model is more comprehensive than Young’s view.

##### 4.2.3.3 Stages model

Grohol’s stages model suggests that net disorder is merely a temporary state of fascination for new internet users (Wang et al., 2023). This is the first stage of using the internet. With help, users can easily break away from the fascinated state and enter the awakening second stage, where they start to avoid the risks of internet disorder. When they can balance internet use with other activities, they reach the balanced third stage. The model believes that everyone can eventually reach this balance.

##### 4.2.3.4 Biopsychosocial integrated model

Beard’s integrated model attempts to provide a systematic explanation for the formation of online addiction from the perspectives of biology, psychology, and sociology (Bolton, 2023). Biological factors include physiological and neurobiological variations that may lead to addictive behaviors. Psychological factors involve poor emotional states, loneliness, depression, and other emotions that can easily lead to internet disorder. Social factors include adverse family environments and poor interpersonal relationships, which can make individuals use the internet as a way to resolve conflicts and alleviate anxiety.

##### 4.2.3.5 Compensatory hypothesis

Compensatory hypothesis is the psychological theory suggesting that individuals may develop strengths in certain areas to compensate for perceived or actual weaknesses in others, thereby maintaining self-esteem and overall functional balance (Bäckman and Dixon, 1992). During the developmental process of adolescents, if their psychological development is hindered, they may compensate through behaviors such as online gaming or socializing. This is known as “compensatory loss.” If the compensatory behavior is healthy and “constructive,” the internet use can return to normal; if it is pathological and the process deviates or interrupts, it can lead to technology addiction. When individuals have an insecure attachment style, they have a higher rejection sensitivity and a lower psychological capital to cope with external environmental challenges. According to the social compensation hypothesis, individuals with high rejection sensitivity may perceive their offline social networks as insufficient and may compensate by more extensively using online social networking sites (Valkenburg and Peter, 2001).

### 4.3 Mediating factors related to internet addiction among college students with social media dependency

#### 4.3.1 From psychological mediating factors

##### 4.3.1.1 Anxiety

According to research, anxiety is prevalent among college students, potentially related to significant academic pressure, personal life troubles, and interpersonal stress. The level of anxiety among college students is positively correlated with the time spent on social media (Baltacı, 2019). Excessive time spent on social media, particularly among young people, can lead to more severe anxiety symptoms (Vannucci et al., 2017). Overuse of social networking is also time-consuming, as it deprives individuals of ordinary group experiences with peers, leading to loneliness, isolation, and anxiety (Chi et al., 2019). Furthermore, internet addiction is significantly correlated with anxiety in adolescents (Gholamian et al., 2019), and using multiple social media platforms is associated with higher levels of anxiety (Primack et al., 2017). Additionally, Facebook addiction is related to anxiety in college students (Zaffar et al., 2015). Some studies suggest that college students with anxiety disorders prefer online communication because they find it difficult to interact face-to-face (Kuss and Griffiths, 2011). This may lead to an increase in the time they spend online, which in turn increases the incidence of online addiction. When using social media, the regular checking of social media can lead to FOMO (fear of missing out) anxiety, which further intensifies the compulsion to check social networks (LeBourgeois et al., 2017), exacerbating web addiction. There is a close connection between FOMO and internet disorder, with individuals experiencing high levels of FOMO often suffering from more severe technology addiction issues. This indicates that anxiety plays a mediating chain role between social media use and net addiction.

##### 4.3.1.2 Self-esteem

The concept of self-esteem reflects an individual’s global evaluation of themselves. Empirical research has highlighted the connections between self-esteem and subjective well-being (Pandey et al., 2021), life satisfaction (Błachnio and Przepiorka, 2016), happiness (Du et al., 2017), and mental health (Sowislo and Orth, 2013). Individuals with lower life satisfaction, happiness, and subjective well-being often adopt negative coping strategies, experience more negative emotions, and are prone to depression and psychological dysfunction. The theory of compensation for the lack of internet disorder suggests that it is precisely because college students’ various needs in real life are disrupted and unsatisfied, leading to a surge in negative emotions and feelings of frustration, that they turn to the internet to seek satisfaction and a sense of achievement, exhibiting “pathological compensation.” The direct effect of this on online addiction is not significant, but rather it indirectly affects internet addiction through self-esteem. Previous studies have indicated a significant negative correlation between net disorder and self-esteem among college students (Greenberg et al., 1999). College students are in a period of “psychological weaning,” facing various problems in real life. If these issues are not effectively resolved for a long time, their self-esteem may be somewhat damaged. When self-esteem is difficult to satisfy in reality, people tend to seek fulfillment in the illusory online world, increasing the propensity for online addiction. Furthermore, research suggests that lower self-esteem may be a risk factor for various forms of addiction (Gori et al., 2023; Gori et al., 2021), including issues related to social media use (Weaver and Swank, 2021; Kircaburun et al., 2019), indicating that self-esteem plays a complete mediating role between social media use and online addiction. Similarly, self-esteem has been found to be negatively correlated with FOMO (Weaver and Swank, 2021), which may be a mediating factor in this relationship. The lower the self-esteem of college students, the more prone they are to cyber addiction. Low self-esteem and unrealistic optimism both have predictive effects on internet addiction (Kim and Davis, 2009). Self-esteem has a self-regulatory function, and individuals with higher levels of self-esteem can effectively cope with life stresses and reduce the occurrence of negative behaviors. Therefore, when college students have higher life satisfaction, they will have positive experiences, internalize and enhance their self-esteem, enabling them to more correctly understand and use the internet, thus preventing and reducing online addiction.

##### 4.3.1.3 Depression

Depression is a common mental disorder characterized by sadness, feelings of worthlessness, and a loss of interest or pleasure in performing activities (Yuan et al., 2024). Currently, research on the potential impact of Problematic Social Media Use (PSMU) on depression is expanding, with conflicting findings (Kross et al., 2013), and some studies have found an opposite relationship. For example, social media can provide social support, increase life satisfaction, and thereby reduce depressive symptoms (Bányai et al., 2017; Salmela-Aro et al., 2017). Conversely, many studies have found an association between PSMU and depressive symptoms in college students (Bányai et al., 2017). The connection between depression and social media use can be explained by the neglect of basic life aspects by those who overuse social media. Significantly increased time spent on social networking sites may lead to reduced face-to-face social interactions, disrupted sleep patterns, and decreased physical activity (Pollet et al., 2011), all of which can contribute to the manifestation of depressive symptoms. Although it is easy to interact with a wider audience, online relationships through social networks are considered to be of lower quality than daily face-to-face communication and are associated with higher levels of loneliness (Online et al., 2014), thus the use of social media is thought to contribute to the onset of depression. Multiple previous studies have suggested that there may be a bidirectional relationship between internet disorder and depression (Chun et al., 2017; Khan et al., 2017). On one hand, depression may be a trigger for addictive behaviors (Starcevic and Khazaal, 2017; Laconi et al., 2017). Some research suggests that using social media, online gaming, etc., may be one of the escape strategies to alleviate depression, so using the internet can, to some extent, alleviate depressive symptoms, including feelings of low mood (Wang et al., 2017). Correspondingly, depression also increases the likelihood of technology addiction (Kuss and Griffiths, 2017; Fried et al., 2017). On the other hand, the process of internet addiction, accompanied by withdrawal symptoms and impaired social functioning, also increases the likelihood of depression (Zadra et al., 2016). In summary, the use of social media increases the probability of depression, and depression may lead to net addiction behaviors, with depression being an intermediary factor between social media use and internet addiction.

#### 4.3.2 From social barriers mediating factors

The rapid development of social media has largely broken down the traditional boundaries of social interaction, enabling us to easily communicate with people around the world without leaving our homes, even to establish connections with strangers. However, the expansion of social circles and the ease of communication do not necessarily mean that modern individuals can obtain more emotional support from frequent online social interactions. “Loneliness” remains a prominent issue in modern society, and it does not seem to have been alleviated with the widespread adoption of social media. In contrast, Sherry Turkle, a social psychologist at MIT in the United States, proposed the concept of “solitude together” in 2011 (Turkle, 2011). She believes that the extensive use of social media has, to some extent, exacerbated people’s feelings of loneliness, as new communication technologies are changing the real-world bonds between individuals. Social media may appear to facilitate interpersonal connections but actually lead to greater emotional distance between people. With little research on the subject, it has been found that the use of social media is directly related to lower social support, greater social isolation, and loneliness (Asa et al., 2020). Studies have shown that excessive use of social media may reduce perceived social support. In fact, a longitudinal study of 221 American college students found a correlation between an increase in problematic social media use and a decrease in tangible social support (Lapierre and Zhao, 2021). Social media users have a stronger communication need than those who do not use social networks, and when they extensively use social media, they may inadvertently replace real-world interactions with virtual relationships, which may increase perceived social isolation (Meshi et al., 2020). Excessive use of social media also reduces face-to-face interactions and diminishes the depth and quality of interactions between family members and friends, potentially creating or increasing psychological distance between individuals, thereby negatively impacting the relationships between college students and their families and friends, leading to feelings of loneliness (Lin et al., 2021; Mk et al., 2021). Heavy users or individuals with problematic social media use may feel more lonely and less satisfied with their interpersonal needs (Gong et al., 2021). Many domestic and international studies have found that digital addiction tendency is closely related to negative social emotions such as emotional social loneliness. When people feel lonely, they may seek social support online, and college students are more likely to use the internet to express their opinions and release emotions, leading to an increase in the incidence of internet addiction. Studies have shown that perceived social support has a positive impact on individuals (Gutman and Midgley, 2000), and college students who receive positive support from parents, society, and teachers have good academic and social performance. Those who cannot obtain or obtain insufficient social support are more likely to meet their interpersonal needs and establish alternative social relationships through the internet, thereby increasing the risk of internet disorder. In summary, social media use can directly influence the tendency toward internet addiction and can indirectly influence the tendency toward online addiction through emotional social loneliness as a mediating variable.

#### 4.3.3 From COVID-19 pandemic mediating factors

As COVID-19 has become a health, economic, and social emergency (Mallah et al., 2021) and a unique disaster (Peleg et al., 2021), every aspect of people’s lives has undergone significant changes (Lee et al., 2022). Studies have shown that the detection rate of problematic online behavior among adolescents during the pandemic is significantly higher than the average detection rate, indicating the need for a deep analysis of problematic online behavior among adolescents during the pandemic.

First, from the perspective of individual behavior, during the COVID-19 pandemic, a large amount of offline teaching shifted to online platforms (Zurlo et al., 2020). Social media helped adolescents engage in educational activities online and maintain connections with peers during the period of isolation, becoming an important means to cope with continued learning problems and combat loneliness, thereby helping them obtain positive emotions. However, research also found that the pandemic led to an increase in anxiety and depression levels among adolescents, which in turn increased problematic social media use (Chen et al., 2021). If an individual is mildly infected with the SARS-CoV-2 virus, it can lead to dysregulation of multiple neuroglial cells and myelin sheaths, manifesting as impaired attention, information processing speed, memory, and executive functions, commonly referred to as “COVID fog.” It is speculated that the pandemic-induced “premature aging” of the brain and cognitive sequelae may weaken adolescents’ emotional regulation abilities and executive functions, making it more difficult to resist online temptations and potentially leading to internet addiction. Second, from the perspective of changes in personal environments, the dramatic changes in daily routines due to the pandemic, such as being confined to the home, the deprivation of outdoor activities, and the increase in stress in both learning and life (Hakami et al., 2021), coupled with the lack of online psychological intervention measures for major public health emergencies, have made social media a common way for adolescents to release emotions and alleviate stress. The relief of mental health problems caused by excessive internet use further strengthens internet use behaviors, potentially leading to excessive and uncontrolled internet use. These factors may lead to excessive social media use and potential social media addiction among college students during the COVID-19 pandemic.

Third, from the perspective of the middle system and the external system, namely families and schools. The changes in daily routines caused by the pandemic have led to more parental monitoring and scolding of adolescents, reducing parent–child closeness and increasing anger, arguments, neglect, and disgust (Jiang et al., 2023). Additionally, the pandemic has also increased parental anxiety and depression, leading to more parent–child conflicts and further increasing adolescents’ anxiety and depression, making them more prone to addictions to mobile phones and other electronic products. The prolonged online teaching and online school-home communication during the pandemic have raised the work requirements and increased the workload for teachers, leading to more job burnout (Chen et al., 2020), hindering teachers from providing more support for students, thus increasing the risk of problematic online behavior among Chinese adolescents during and after the pandemic. In summary, the COVID-19 pandemic has led to excessive use of social media and has increased the incidence of online addiction.

### 4.4 Intervention and prevention measures for college student internet addiction related to social media dependence

#### 4.4.1 Cognitive therapy

Studies have shown that group counseling and Cognitive Behavioral Therapy (CBT) are the most widely used methods for reducing Internet Addiction (IA) (Liu et al., 2017). Group counseling aims to provide group psychological support and guidance, with helpers forming target groups based on the similarity of participants’ problems. Huang et al. conducted a meta-analysis of 11 group counseling intervention studies for Chinese college students with IA and found that group counseling can effectively improve IA students’ communication skills and reduce their obsessive-compulsive and anxiety symptoms (Huang et al., 2015). Another meta-analysis found that group counseling intervention programs are effective in reducing IA in four aspects: time management, interpersonal relationships and health issues, tolerance, and compulsive online use (Liu et al., 2017). The cognitive-behavioral model points out that maladaptive cognitions are the fundamental cause of pathological internet use. Maladaptive cognitions include distorted cognitions of oneself and the external world, such as self-doubt, low self-efficacy, negative self-esteem, or the belief that respect can only be obtained online (Davis, 2001). Additionally, the individual’s psychopathology (e.g., depression and social anxiety) and stressors from the external environment (e.g., family and school) are considered to be remote causes of IA (Davis, 2001). IA CBT intervention programs based on the cognitive-behavioral model include identifying maladaptive cognitions, triggers for addictive emotions and behaviors, restructuring cognitions, learning appropriate behavioral skills, and seeking social support. Meta-analyses have shown that CBT programs have positive effects on IA patients in terms of depression, anxiety, aggression, somatization, social anxiety, fear anxiety, paranoid ideation, and psychoticism (Liu et al., 2017).

#### 4.4.2 Behavioral therapy

Physical exercise can improve an individual’s attentional bias. Attentional bias refers to the individual’s selective attention to information. Studies have shown that internet addicts have a stronger attentional bias toward negative information stimuli compared to positive and neutral information (Hu et al., 2020). Eye-tracking results also indicate that internet addicts generally show a pattern of unbiased attention and overall avoidance toward positive words. Physical exercise can have a positive impact on an individual’s attentional processes, especially selective attention. Substantial evidence suggests that exercise can increase an individual’s ability to identify addiction-related cues in the early stages, enhance the regulation of attentional resources, and convert excessive attentional engagement with internet-related cues into attentional inhibition, helping the individual detach from internet-related cues. Furthermore, exercise can regulate the fluctuations of N170 and P2 components in the brain that are related to attention control and error processing (Zhao et al., 2015), reducing the amplitude of N170 and P2 components induced by internet-related cues, reducing attentional bias and cognitive control conflicts caused by internet cues, and helping the individual better control addictive internet behaviors. Exercise has been widely studied as an alternative or adjunctive treatment for net addiction due to its psychological benefits, such as reducing depression, anxiety, and anger, and improving mood (Zhang, 2012; Hassmen et al., 2000), as well as its physical benefits, such as enhancing cardiovascular function, promoting blood circulation, improving immune response, and neural function. A meta-analysis concluded that exercise interventions can significantly reduce internet addiction (Liu et al., 2017), and a study showed that exercise can significantly reduce internet use time and the severity of internet disorder (Kocak, 2018). Therefore, we believe that exercise-based intervention may be an effective method for preventing, alleviating, and even eliminating digital addiction.

#### 4.4.3 Other treatments

In fact, there have been cases of using anti-anxiety and anti-depressant drugs to treat online addiction several years ago. We can use anti-anxiety and anti-depressant psychiatric drugs to alleviate the addicts’ negative emotions and create favorable conditions for psychological treatment. Currently, drug intervention combined with psychological counseling has been widely used as a comprehensive treatment approach in clinical practice.

Some patients may turn to addictive internet use due to a lack of social support in real life. They use some interactive functions of the internet as substitutes for real-life social support. During treatment, it is also important to consider these factors and provide a safe, relatively free, and relaxed environment for them. Encouraging them to participate in interest groups can help them meet friends with similar backgrounds, reducing their reliance on internet interactions.

### 4.5 Advantages and limitations

The WOS database is a globally authoritative citation database that includes a series of core journals with the most academic impact in various disciplines. To some extent, these documents can reflect the global trends in the forefront of specific disciplines or fields. By examining the quantity and quality of scientific materials produced, we can objectively evaluate the current state and level of advancement of science. This study, based on the WoS core database, employed bibliometric methods to investigate the impact of social media on college student net addiction over the past decade. The use of multiple vivid knowledge maps and tables illustrated the development trajectory of the field, identified core producers, revealed the scientific collaboration network between authors and countries, and clarified the interdisciplinary nature of the research. This study also analyzed and discussed the research hotspots in the field, providing insights for future research directions and methods.

However, this study also has some limitations. Firstly, the use of the WoS core database as a source of literature may introduce significant differences due to the varying results from different databases. If the literature was obtained from other databases, more citation or article changes may have been observed, limiting the generalizability of the research results. Secondly, we only analyzed journal articles and reviews, excluding other relevant publications in the field, such as book chapters and conference proceedings. Thirdly, we only collected English literature from the WOS database over the past 10 years through specific search strings, not including all the literature in the field. This may have affected the comprehensiveness of this study. Due to the above limitations, when analyzing hot trends, it may not cover other databases and literature types, misjudge the importance of topics or miss emerging trends, and it is difficult to deeply analyze the reasons for the data from a multi-disciplinary, cultural and methodological perspective, reducing the reliability and persuasiveness of the conclusions. Future research can integrate multiple representative databases, such as PubMed, Scopus, etc., and develop a comprehensive search strategy to cover different literature types, and multiple languages. At the same time, professional literature management and analysis tools are used to integrate and analyze multi-source data to reduce the limitations of a single database, literature type and language, and ensure that the research results are more comprehensive and universal. Additionally, while this study provides a comprehensive bibliometric analysis of social media dependence and internet addiction among college students, it is important to acknowledge the inherent challenges of achieving significant breakthroughs in a well-researched area. The field has seen extensive exploration over the past decade, making it difficult to introduce entirely novel perspectives. However, our work contributes by systematically mapping the research landscape, identifying emerging trends, and highlighting interdisciplinary connections that may inspire future studies. Future research could explore more granular analyses, such as longitudinal studies or cross-cultural comparisons, to uncover deeper insights into the mechanisms of internet addiction and its mediating factors.

## 5 Conclusion

Overall, the results of this study have revealed research trends over the past decade and are expected to provide a better understanding of the scientific patterns between social media and internet addiction among international college students. Firstly, researchers’ interest in the intrinsic connections and interactions between social media and digital addiction among college students has increased over the past decade, indicating that this field still needs to be further developed. Secondly, the research in this field is relatively interdisciplinary, and we hope that researchers from different fields can participate in this research in the future. Together, they can contribute to addressing the addiction behavior problems of college students worldwide and reduce the incidence of internet disorder. Thirdly, there are numerous mediating factors in social media and cyber addiction among college students, such as mental health issues, social barriers, and pandemics. Therefore, we need to expand the scope of research to understand as many specific mediating factors as possible, so that we can provide corresponding strategies for the prevention and control of internet addiction among college students. Fourthly, over the past decade, researchers have conducted certain studies on the external and internal factors between social media and digital addiction among college students and have achieved some results. However, research on the internal factors, such as the complex pathogenesis and pathology, is not yet deep enough. Therefore, it is necessary to conduct more in-depth research to explore the underlying mechanisms.

Although the findings of this study are based on college students, their generalizability to other populations may be limited due to demographic and contextual differences. However, we believe these findings provide a valuable starting point for the research on broader populations, which could contribute to a deeper understanding of the relationship between social media dependence and internet addiction across different age groups and cultural contexts. Future research could explore the mechanisms underlying these factors in different populations, offering a more comprehensive understanding of social media dependence and internet addiction. In summary, this study offers valuable guidance and reference points for researchers to contemplate and define future research directions. By delineating the boundaries of this field, it reduces the effort required for exploration and lays a solid theoretical foundation for subsequent studies in this area.

## Data Availability

The raw data supporting the conclusions of this article will be made available by the authors, without undue reservation.
